# The association between *vacA* or *cagA* status and eradication outcome of Helicobacter pylori infection: A meta-analysis

**DOI:** 10.1371/journal.pone.0177455

**Published:** 2017-05-11

**Authors:** Dan Wang, Qiuping Li, Yuehua Gong, Yuan Yuan

**Affiliations:** Tumor Etiology and Screening Department of Cancer Institute and General Surgery, the First Affiliated Hospital of China Medical University, and Key Laboratory of Cancer Etiology and Prevention (China Medical University), Liaoning Provincial Education Department, Shenyang, China; University Hospital Llandough, UNITED KINGDOM

## Abstract

**Background:**

*H*. *pylori* virulence factors, especially *vacA* and *cagA* are important in gastroduodenal disease pathogenesis and affect cure rates. This meta-analysis aimed to clarify the association between *vacA* or *cagA* status and eradication outcome of *H*. *pylori* infection.

**Methods:**

A literature search was performed using electronic databases to identify studies. Twenty-six prospective studies were determined eligible. Meta-analytical techniques were conducted to calculate eradication rates and pooled relative ratios (RR).

**Results:**

The eradication rate was greater approximately 10% in *vacA* s1 compared with *vacA* s2 infected patients, and the pooled RR was 1.164 (95%CI: 1.040–1.303, *P* = 0.008). A significant association existed between *vacA* s1 and higher eradication rates in Europe (RR: 1.203, 95%CI: 1.003–1.442, *P* = 0.046) and Asia (RR: 1.187, 95%CI: 1.028–1.371, *P* = 0.020), in triple therapy patients (RR: 1.175, 95%CI: 1.012–1.365, *P* = 0.035). Eradication rates were similar for *vacA* m1 and m2 genotypes (RR: 0.981, 95%CI: 0.891–1.080, *P* = 0.690), whereas they were higher by approximately 8% in *cagA*-positive compared with *cagA*-negative infected patients, with a pooled RR of 1.094 (95%CI: 1.025–1.168, *P* = 0.007). A significant association existed between *cagA*-positive and increased eradication rates in Europe (RR: 1.138, 95%CI: 1.000–1.295, *P* = 0.049) and Asia (RR: 1.118, 95%CI: 1.051–1.190, *P*<0.001), in using PCR (RR: 1.232, 95%CI: 1.142–1.329, *P*<0.001) and protein chips (RR: 1.200, 95%CI: 1.060–1.359, *P* = 0.004), in triple therapy patients (RR: 1.090, 95%CI: 1.006–1.181, *P* = 0.034).

**Conclusions:**

Evidence indicates that infection with *vacA* s1, *cagA*-positive strains, but not *vacA* s2, *cagA*-negative, is more conducive to *H*. *pylori* eradication.

## Introduction

*Helicobacter pylori* (*H*. *pylori*) is among the most common pathogenic microorganisms in the world and is involved in the pathogenesis of gastritis, gastroduodenal ulcers, gastric cancer and other diseases[1]. At present, *H*. *pylori* eradication therapy for symptomatic patients is universally recognized. A number of prospective cohort studies suggest that *H*. *pylori* eradication is beneficial to patients by preventing the progression of gastric diseases[2, 3]. With the widespread application of eradication therapy, eradication rates have continued to decline steadily over the last decade. However, how to successfully eradicate *H*. *pylori* is still a concern worldwide.

*H*. *pylori* eradication is affected by a number of variables. In addition to host factors, bacteria themselves are also widely believed to play a crucial role, and more research is being conducted on bacterial mutation, biofilm formation, efflux pumps as well as other factors. Additionally, certain virulence factors secreted by *H*. *pylori*, which are helpful in bacterial colonization, induction of inflammation, immune evasion and cancer promotion[4], may also affect outcomes of *H*. *pylori* eradication[5]. Vacuolating cytotoxin A (*VacA*) and cytotoxin-associated gene A (*CagA*) are two important virulence factors of *H*. *pylori*. *vacA* gene-encoded vacuolating toxins can induce apoptosis, inhibit T-cell activity and avoid clearance by host immunity[6]. DNA sequence analysis has revealed that the VacA protein has a mosaic structure comprising allelic variations in the signal (s) and mid region (m), each having two alleles (s1/s2, m1/m2) with different biological activities. The s1 and m1 regions have been associated with peptic ulcer and an increasing risk of gastric cancer[7]. Furthermore, some reports noted that *vacA* genotypes have different effects on *H*. *pylori* eradication. For example, Van Doorn et al.[8] pointed out that *vacA* s1 strains had higher *H*. *pylori* eradication rates compared with *vacA* s2 strains, but López-Brea et al.[9] indicated there was no difference between *vacA* s1 and *vacA* s2 strains. Similarly, for *vacA* m1 and *vacA* m2, Niu et al.[10] reported that eradication rates were higher with *vacA* m1 strains. However, Chaudhuri et al.[11] drew the opposite conclusion. Another virulence factor closely related to *H*. *pylori* pathogenicity is *cagA*. There is also inconsistency in the relationship between *cagA* and *H*. *pylori* eradication. For example, a literature search[8] showed that cure rates in patients infected with *cagA*-positive strains were significantly higher than in patients infected with *cagA*-negative ones, whereas Huang et al.[12] came to the opposite conclusion. Studies by Magalhaes et al.[13] and Baryshnikova et al.[14] showed that *cagA*-positive or *cagA*-negative strains did not affect eradication rates.

Thus, virulence factors *vacA* and *cagA* are not only closely related to pathogenicity but also may be among the main aspects influencing *H*. *pylori* eradication. However, these results remain controversial. Clarification of the correlation between virulence factors and eradication therapy will aid in the rational selection of eradication regimens and in the prediction of eradication outcomes. Therefore, we undertook a systematic review to evaluate the effect of virulence factor *vacA* and *cagA* status on eradication treatment.

## Materials and methods

### Identification and eligibility of relevant studies

Electronic databases of PubMed, Embase, Cochrane Library, Web of Science and Wanfang Data, Chinese National Knowledge Infrastructure (CNKI), China Biology Medicine disc (CBMdisc), and China Science and Technology Jouranl Database (VIP), were systematically searched using the terms, “*vacA*”, “*cagA*”, “*H*. *pylori*/*Helicobacter pylori*”, and “eradication/therapy/treatment”. The corresponding Chinese terms were used when searching Chinese databases. Furthermore, references that were cited in each included study were also searched manually to identify potential, additional relevant studies. If the information provided in the article was not sufficiently clear, we contacted the author for detailed raw data. The last search date was October 1, 2016.

### Inclusion and exclusion criteria

Studies included in this meta-analysis must meet the following inclusion criteria: studies published in English or in Chinese; studies investigating the association of *vacA* or *cagA* status for successful eradication of *H*. *pylori* infection; studies with sufficient raw data for estimating RR and their 95% confidence interval (CI). Exclusion criteria: reviews or meta-analyses; animal or cytology experiments; duplicate publications; studies not involving *vacA* or *cagA*; and studies published neither in English nor Chinese; no data of eradication cases to *vacA* or *cagA* status.

### Data extraction

Two authors (Dan Wang and Qiuping Li) extracted the data independently from the included studies. Any conflicts were resolved after discussion, and consensus was finally reached on all extracted data. The following information was extracted from each study: first author, year of publication, country, region, disease, eradication detection method, detection methods for *vacA* and *cagA* status, treatment, therapeutic regimen, and numbers of successful and failed eradications according to *vacA* and *cagA* status.

### Quality assessment

The Newcastle–Ottawa scale (NOS) with eight items was used to estimate the validity of the included studies[15]. We evaluated the studies on a nine star scale based on selection (four stars maximum), comparability (two stars maximum) and outcome (three stars maximum). NOS scores of 1–3, 4–6 and 7–9 were considered low, medium and high quality, respectively.

### Statistical analysis

The statistical analysis was carried out by Stata software (Version 11.0; StataCorp, College Station, TX, USA). The eradication rate was calculated by per-proptocol (PP) analysis. The pooled *H*. *pylori* eradication rates were assessed via a random-effects model. Cumulative RRs and the corresponding 95% CIs were used to measure the strength of associations between the *vacA* or *cagA* status and eradication of *H*. *pylori*. *P* value<0.05 was considered statistically significant. Heterogeneity across the studies was assessed using a Q statistic (*P*<0.10 indicates significant heterogeneity between studies) and an I-squared (I^2^) value[16].A fixed-effects model using the Mantel–Haenszel method was performed when Heterogeneity between studies was not significant[17]. Otherwise, a random-effects model based on the DerSimonian and Laird method was used[18]. A sensitivity analysis was performed to explore heterogeneity when significant heterogeneity was indicated. Subgroup analysis was used to explore the effect of region and peptic ulcer disease (PUD) with non-peptic ulcer disease(NPUD), the detection method of eradication, therapeutic regimen. Moreover, publication bias was evaluated quantitatively using Begg’s[19] and Egger’s tests[20]. Significant publication bias was indicated if *P* value<0.05.

## Results

### Characteristics of the included studies

**Fig 1:**
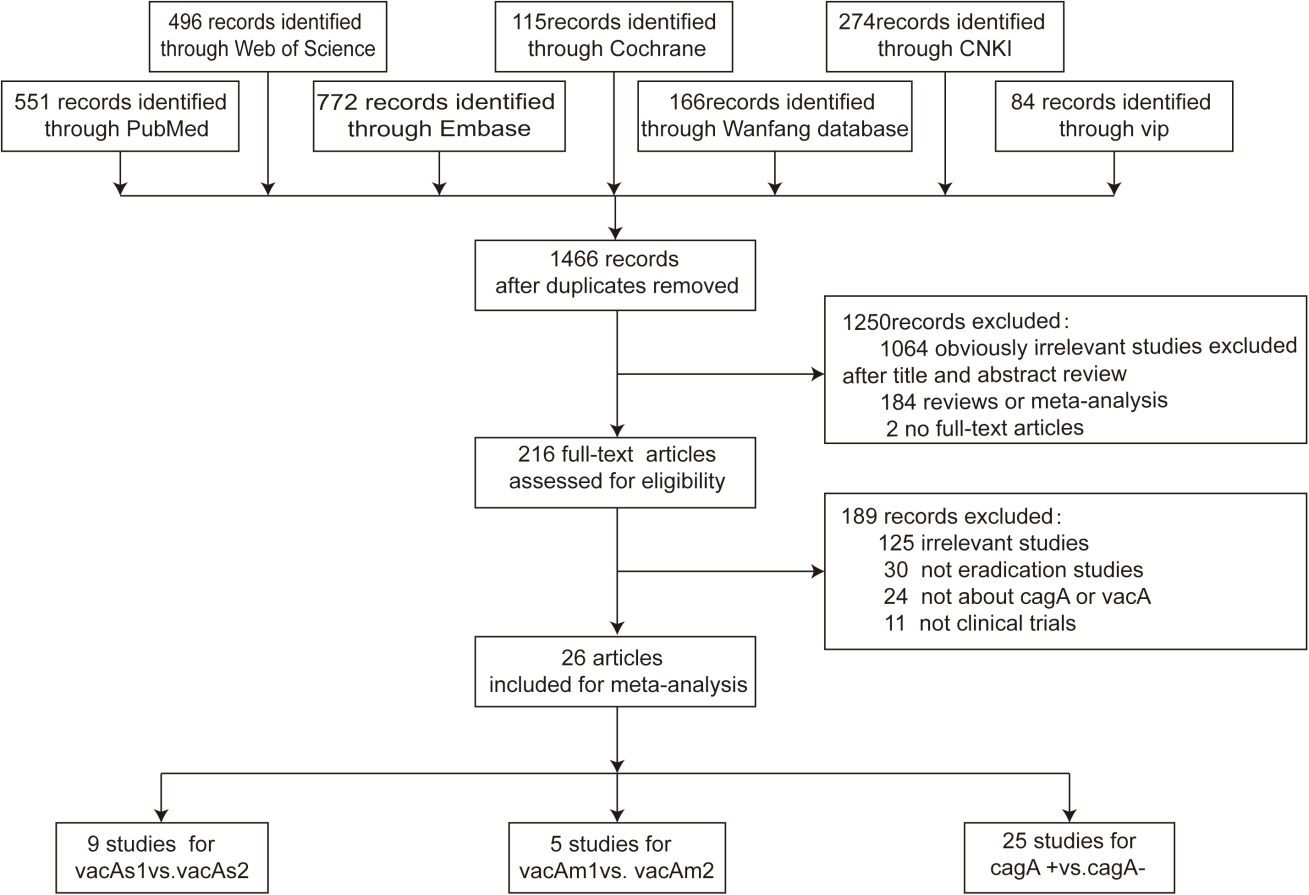
Flow chart of literature search and study selection.

This meta-analysis was organized according to the PRISMA statement (S1 Table). A systematic search of Chinese and English electronic databases yielded 1466 citations after removal of duplicates. The flow chart of included studies is summarized in Fig 1. By screening titles and abstracts, we excluded 1064 citations that were apparently irrelevant, 184 that were reviews or meta-analyses and two that were not full-text articles. After reviewing the full texts of the remaining 216 citations, we removed 125 that were not relevant to this analysis, 30 that were not eradication studies, 24 that were not about *cagA* or *vacA* and 11 that were not clinical trials. Finally, 26 studies that met the inclusion criteria were selected for the meta-analysis. The characteristics of the 26 included prospective studies are presented in Table 1.The NOS results indicated that all the included studies were at an high level of quality with scores ranging from 7 to 8, because some studies did not provide specific selection criteria of control group, some studies only include one kind of gastric disease and most studies did not fully consider the control factor for the comparability of cases and controls such as age and sex. Detailed results for NOS quality assessment were summarized (S2 Table).

**Table 1:** Characteristics of selected studies in this meta-analysis.

Author	Year	Country	Region	Disease	Detection method of eradication	Method	Treatment	Therapeutic regimen	Case			Control			
									Eradication	Faliure	Eradication rate	Eradication	Faliure	Eradication rate	
**For *vacA*s1/s2**															
López-Brea[9]	1999	Spain	South American	NPUD、PUD	Serology、RUT、Histology、Culture、UBT	PCR	BAM,NA	Triple therapy	3	3	0.5000	21	5	0.8077	
Van Doorn[8]	2000	Netherlands	Europen	NPUD、PUD	Histology、RUT、Culture、PCR	PCR	LBTeM,4or5d	Quadruple therapy	56	19	0.7467	11	11	0.5000	
Rudi[31]	2002	Germany	Europen	NPUD、PUD	RUT、PCR、UBT	PCR	LorO+ACorMC,7d	Triple therapy	80	12	0.8700	19	4	0.8261	
Scholte[32]	2002	Netherlands	Europen	NPUD	Histology、RUT、Culture	PCR	OAC,7d	Triple therapy	11	0	0.9995	11	2	0.8462	
He[33]	2002	China	Asian	NPUD、PUD	UBT	PCR	OAC,7d	Triple therapy	85	19	0.8173	4	2	0.6667	
Chaudhuri[11]	2003	India	Asian	PUD	Histology、RUT、Culture	PCR	OAC,10d	Triple therapy	26	16	0.6190	2	3	0.4000	
Russo[30]	2003	Italy	Europen	NPUD、PUD	UBT	PCR	LAC,7d	Triple therapy	67	20	0.7701	9	12	0.4286	
De Francesco[21]	2004	Italy	Europen	NPUD、PUD	UBT	PCR	RA+RTCorRAC,10d	Sequential therapy	40	4	0.9091	46	5	0.9020	
Niu[10]	2014	China	Asian	PUD	Histology、RUT、Cluture、UBT	PCR	LAC,7d	Triple therapy	118	12	0.9077	12	2	0.8571	
**For *vacA*m1/m2**															
Rudi[31]	2002	Germary	Europen	NPUD、PUD	UBT、RUT	PCR	LorO+ACorMC,7d	Triple therapy	44	5	0.8980	55	11	0.8333	
Scholte[32]	2002	Netherlands	Europen	NPUD	Histology、RUT、Culture	PCR	OAC,7d	Triple therapy	5	0	0.9995	16	3	0.8421	
He[33]	2002	China	Asian	NPUD、PUD	UBT	PCR	OAC,7d	Triple therapy	16	4	0.8000	73	17	0.8111	
Chaudhuri[11]	2003	India	Asian	PUD	Histology、RUT、Culture	PCR	OAC,10d	Triple therapy	11	13	0.4583	17	6	0.7391	
**For *vacA*m1/m2**															
De Francesco[21]	2004	Italy	Europen	NPUD、PUD	UBT	PCR	RA+RTCorRAC,10d	Mixed therapy	33	4	0.8919	52	6	0.8966	
**For *cagA*+/-**															
van der Hulst[34]	1997	Netherlands	Europe	NPUD、PUD	Histology、Culture	PCR	OA,14d	Dual therapy	89	33	0.7295	17	16	0.5115	
Greenberg[35]	1999	USA	North America	NPUD	Histology	WB	OC,14d	Dual therapy	22	12	0.6471	8	0	0.9995	
López-Brea[9]	1999	Spain	Europe	NPUD、PUD	Cerology、RUT、Histology、Culture、UBT	PCR	BAM,NA	Triple therapy	6	2	0.7500	18	6	0.7500	
Mao[36]	2000	Vietnam	Asian	PUD	UBT、Histology	ELISA	OACorRAC,10d	Triple therapy	78	5	0.9398	19	2	0.9048	
Van Doorn[8]	2000	Netherlands	Europe	NPUD、PUD	Histology、RUT、Culture	PCR	LBTeM,4or5d	Quadruple therapy	48	11	0.8136	19	19	0.5000	
Lerro[37]	2000	Italy	Europe	NPUD	UBT	WB	OAC,14d	Triple therapy	21	14	0.6000	14	1	0.9333	
Broutet[38]	2001	France	Europe	NPUD	UBT、Histology、Culture	PCR	PAC,NA	Triple therapy	64	20	0.7619	45	27	0.6250	
Saruc[39]	2001	Turkery	Asian	NPUD	Histology、RUT	ELISA	LAC,7d	Triple therapy	111	16	0.8740	41	16	0.7193	
Rudi[31]	2002	Germany	Europe	NPUD、PUD	RUT、PCR、UBT	PCR	LorO+ACorMC,7d	Triple therapy	73	9	0.8902	26	7	0.7879	
Queiroz[40]	2002	Brazil	South America	NPUD、PUD	UBT	PCR	PFC,7d	Triple therapy	68	7	0.9067	15	5	0.7500	
Scholte[32]	2002	Netherlands	Europe	NPUD	Histology、RUT、Culture	PCR	OAC,7d	Triple therapy	10	0	0.9995	13	3	0.8125	
Treiber[29]	2002	Germany	Europe	NPUD、PUD	UBT	PCR	LorR5d+AMC3dor5d	Quadruple therapy	147	14	0.9130	61	9	0.8714	
He[33]	2002	China	Asian	NPUD、PUD	UBT	PCR	0AC,7d	Triple therapy	69	14	0.8313	20	7	0.7407	
DeFrancesco[41]	2002	Italy	Europe	NPUD	UBT	ELISA	RA+RTC,10d	Sequential therapy	27	4	0.8710	24	4	0.8571	
**For *cagA*+/-**															
Chaudhuri[11]	2003	India	Asian	PUD	Histology、RUT、Culture	PCR	OAC,10d	Triple therapy	25	17	0.5952	3	2	0.6000	
Russo[30]	2003	Italy	Europe	NPUD、PUD	UBT	PCR	LAC,7d	Triple therapy	69	22	0.7582	8	11	0.4211	
Xia[42]	2003	Australia	Oceania	NPUD	UBT、Histology	ELISA	OAC,7d	Triple therapy	51	6	0.8947	12	3	0.8000	
De Francesco[21]	2004	Italy	Europe	NPUD、PUD	UBT	PCR	RA+RTCor RAC,10d	Mixed therapy	68	5	0.9315	17	5	0.7727	
Magalhaes[13]	2005	Brazil	South America	NPUD、PUD	Histology、RUT	ELISA	LAC,7d	Triple therapy	30	2	0.9375	25	2	0.9259	
Jianjun[43]	2007	China	Asian	PUD	RUT、Culture	PCR	EAC,7d	Triple therapy	54	4	0.9310	3	5	0.3750	
Cen[44]	2009	China	Asian	NPUD、PUD	UBT	WB	ETCorEAC,7d	Triple therapy	222	27	0.8916	77	19	0.8021	
Wu[45]	2011	China	Asian	NPUD、PUD	UBT	Protein chip	EA+AFC,10d	Sequential therapy	23	1	0.9583	95	23	0.8051	
Huang[12]	2012	China	Asian	NPUD	UBT	ELISA	LACorLAe,7d	Triple therapy	48	20	0.7059	48	15	0.7619	
Huang[12]	2012	China	Asian	NPUD	UBT	ELISA	LACEc,7d	Quadruple therapy	26	4	0.8667	30	3	0.9091	
Cui[46]	2013	China	Asian	NPUD、PUD	UBT	Protein chip	EBAZ,7or21d	Quadruple therapy	68	7	0.9067	24	8	0.7500	

Case:*vacA* s1、*vacA* m1、*cagA*-positve, Contrl:*vacA* s2、*vacA* m2、*cagA*-negative; +: positive,—:negative; NPUD:non peptic ulcer disease, PUD:peptic ulcer disease; UBT:Urea breath test, RUT: rapid urease test assay, PCR:polymerase chain reaction, ELISA:enzyme-linked immuno sorbent assay, WB:western blot; R:rabeprazole, Ra:ranitidine, E:esomeprazole, O:omeprazole,T:tinidazole, C:clarithromycin, A:amoxicillin,B:bismuth,F:furazolidone,Te: tetracycline,Ec:Ecabetsodium,J:josamycin,D:doxycycline.

### Association between *vacA* status and eradication of *H*. *pylori*

#### *vacA* s1 and *vacA* s2 genotypes and eradication of *H*. *pylori*

**Fig 2:**
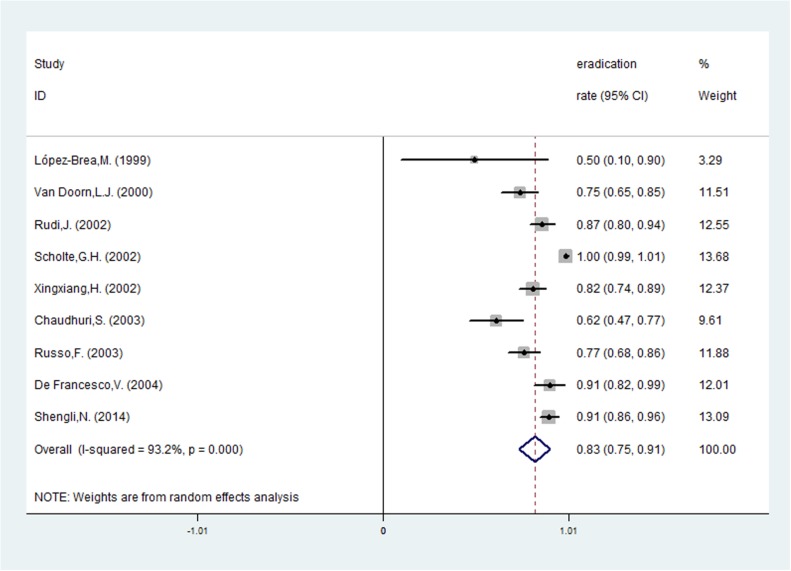
The pooled eradication rate of *H*. *pylori* with *vacA* s1.

**Fig 3:**
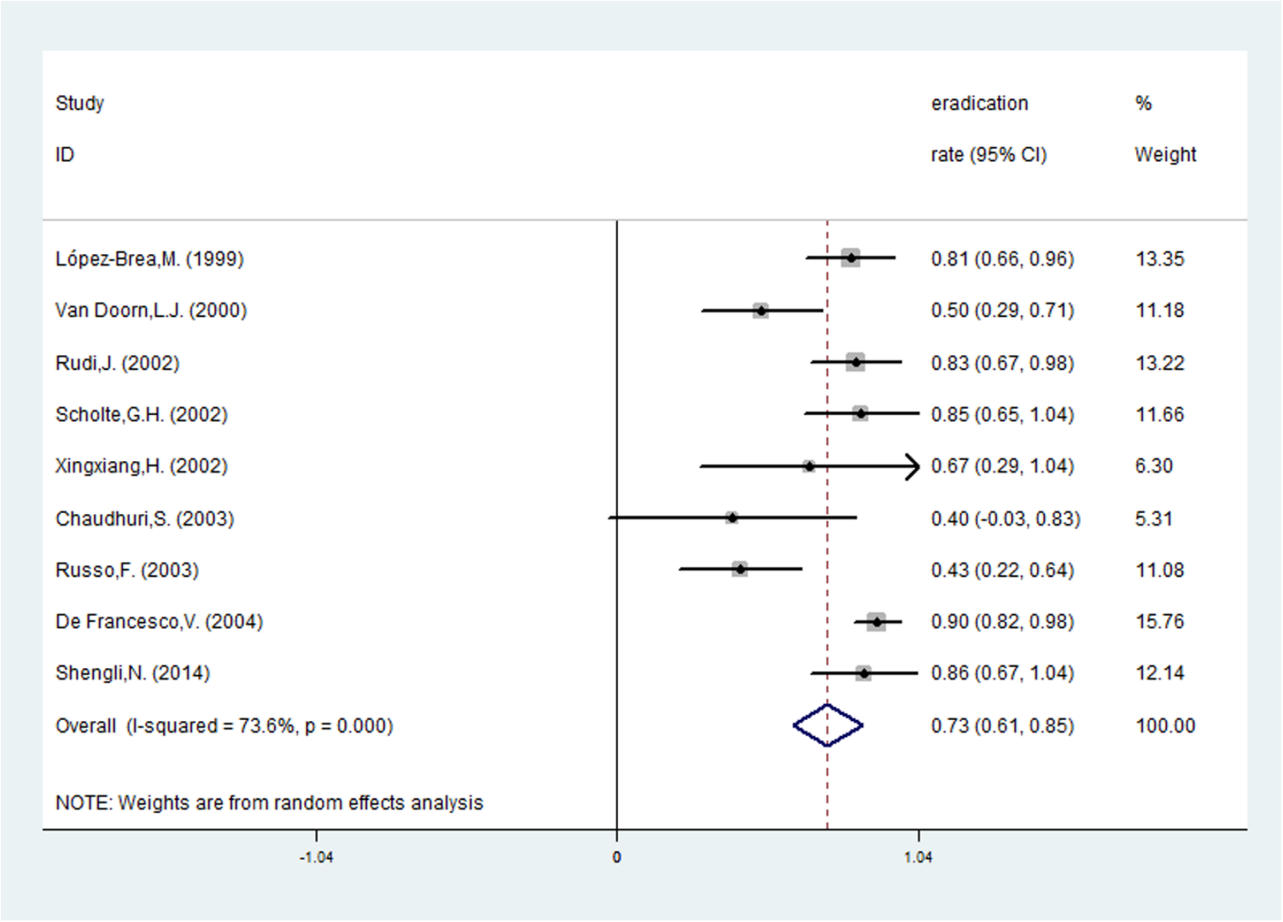
The pooled eradication rate of *H*. *pylori* with *vacA* s2.

**Fig 4:**
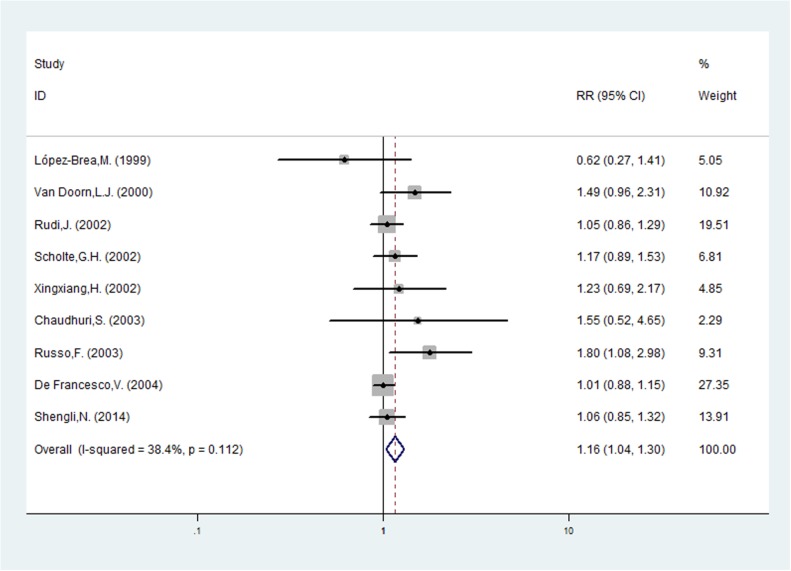
Forest plot of the association between *vacA* s1 and *vacA* s2 and eradication of *H*. *pylori*.

Risk ratios regarding the effects of *vacA* s1 and *vacA* s2 genotypes on *H*. *pylori* eradication rates were available for all nine trials, which included data from 772 patients (591 patients in the *vacA* s1 group and 181 patients in *vacA* s2 group). A fixed-effects model was used because significant heterogeneity was not present (I^2^ = 38.4%, *P* = 0.112). The pooled *H*. *pylori* eradication rate was 83% (95%CI: 75–91%) for *vacA* s1 and 73% (95%CI: 61–85%) for *vacA* s2 (Figs 2 and 3). We found that eradication rates improved by approximately 10% in the *vacA* s1 group compared with the *vacA* s2 group and that the pooled RR was 1.164 (95%CI: 1.040–1.303, *P* = 0.008; Table 2 and Fig 4). Based on these results, we determined that *vacA* s1 strains are more likely to be eradicated by anti-*H*. *pylori* therapy compared with *vacA* s2 strains.

**Table 2:** Meta-analysis result of the association between *vacA* or *cagA* status and eradication of *H*. *pylori*.

Genotype/subgroup		N	Heterogeneity test		Statistical model	Test for overall effect	
			I^2^(%)	*P*_het_		OR(95%CI)	*P*
***vacA*s1/s2**	Overal	9	38.4	0.112	F	**1.164(1.040–1.303)**	**0.008**
	Region						
	Europe	5	24	0.268	F	**1.203(1.003–1.442)**	**0.046**
	Asian	3	59.7	0.042	R	**1.187(1.028–1.371)**	**0.02**
	Treatment						
	Triple therapy	7	17.6	0.296	F	**1.175(1.012–1.36)**	**0.035**
***vacA*m1/m2**	Overal	5	28.1	0.235	F	0.981(0.891–1.081)	0.69
	Region						
	Europe	3	0	0.676	F	1.045(0.949–1.151)	0.372
	Asian	2	67.9	0.078	R	0.819(0.508–1.322)	0.414
***cagA*+/-**	Overal	25	56	<0.001	R	**1.094(1.025–1.168)**	**0.007**
	Region						
	Europe	11	66.6	0.001	R	**1.138(1.000–1.295)**	**0.049**
	Asian	10	29.6	0.172	F	**1.118(1.051–1.190)**	**<0.001**
	South America	2	48.5	0.164	F	1.104(0.953–1.279)	0.186
	Disease						
	NPUD	9	69.8	0.001	R	0.988(0.861–1.134)	0.865
	PUD	3	71.9	0.029	R	1.274(0.664–2.445)	0.467
	Method						
	PCR	13	38.9	0.074	F	**1.232(1.142–1.329)**	**<0.001**
	Protein chip	2	<0.001	0.885	F	**1.200(1.060–1.359)**	**0.004**
	ELISA	7	<0.001	0.449	F	1.048(0.972–1.130)	0.223
	WB	3	89.2	<0.001	R	0.801(0.534–1.203)	0.285
	Treatment						
	Dual therapy	2	93	<0.001	R	0.978(0.414–2.307)	0.959
	Triple therapy	16	46.4	0.022	R	**1.090(1.006–1.181)**	**0.034**
	Quadruple therapy	4	73.2	0.011	R	1.134(0.946–1.360)	0.173
	Sequential therapy	2	48.2	0.165	F	1.114(0.997–1.244)	0.057

Next, we conducted subgroup analyses based on region (European or Asian) and therapy regimen (triple therapy). The regional subgroup analysis showed that for Europe and Asia, pooled RRs were 1.203 (95%CI: 1.003–1.442, *P* = 0.046) and 1.187 (95%CI: 1.028–1.371, *P* = 0.020; Table 2), respectively, regarding the effects of *vacA* s1 compared with *vacA* s2 on eradication rates. The therapy regimen subgroup analysis showed that *vacA* s1 status had higher eradication rates in the triple therapy subgroup (RR: 1.175, 95%CI: 1.012–1.360, *P* = 0.035; Table 2).

### *vacA* m1 and *vacA* m2 genotypes and eradication of *H*. *pylori*

**Fig 5:**
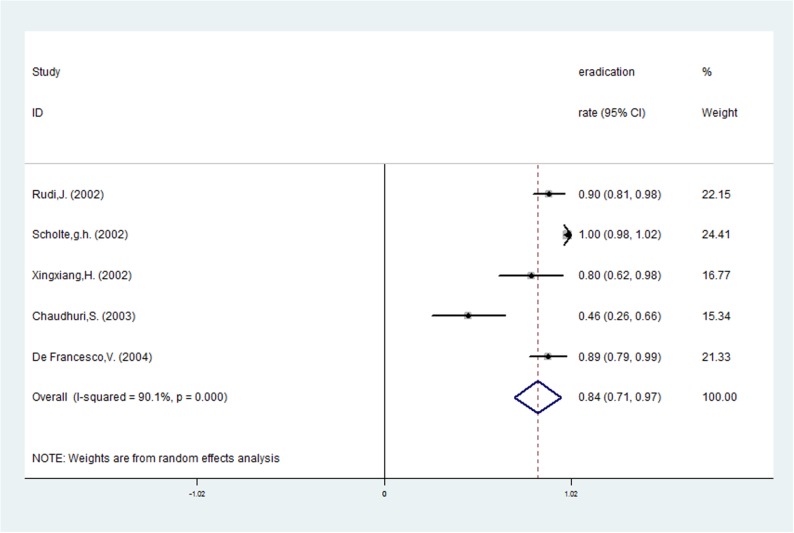
The pooled eradication rate of *H*. *pylori* with *vacA* m1.

**Fig 6:**
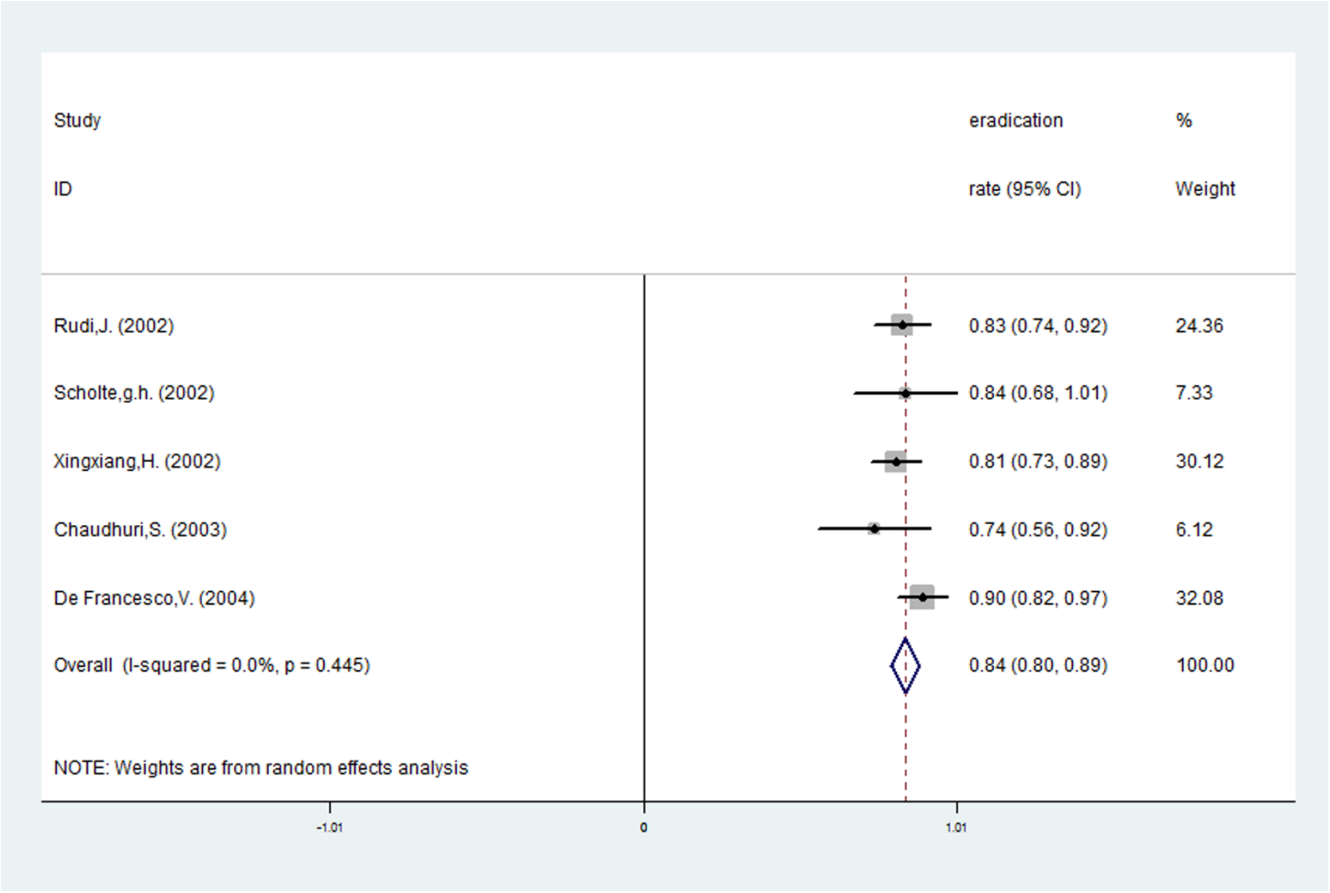
The pooled eradication rate of *H*. *pylori* with *vacA* m2.

**Fig 7:**
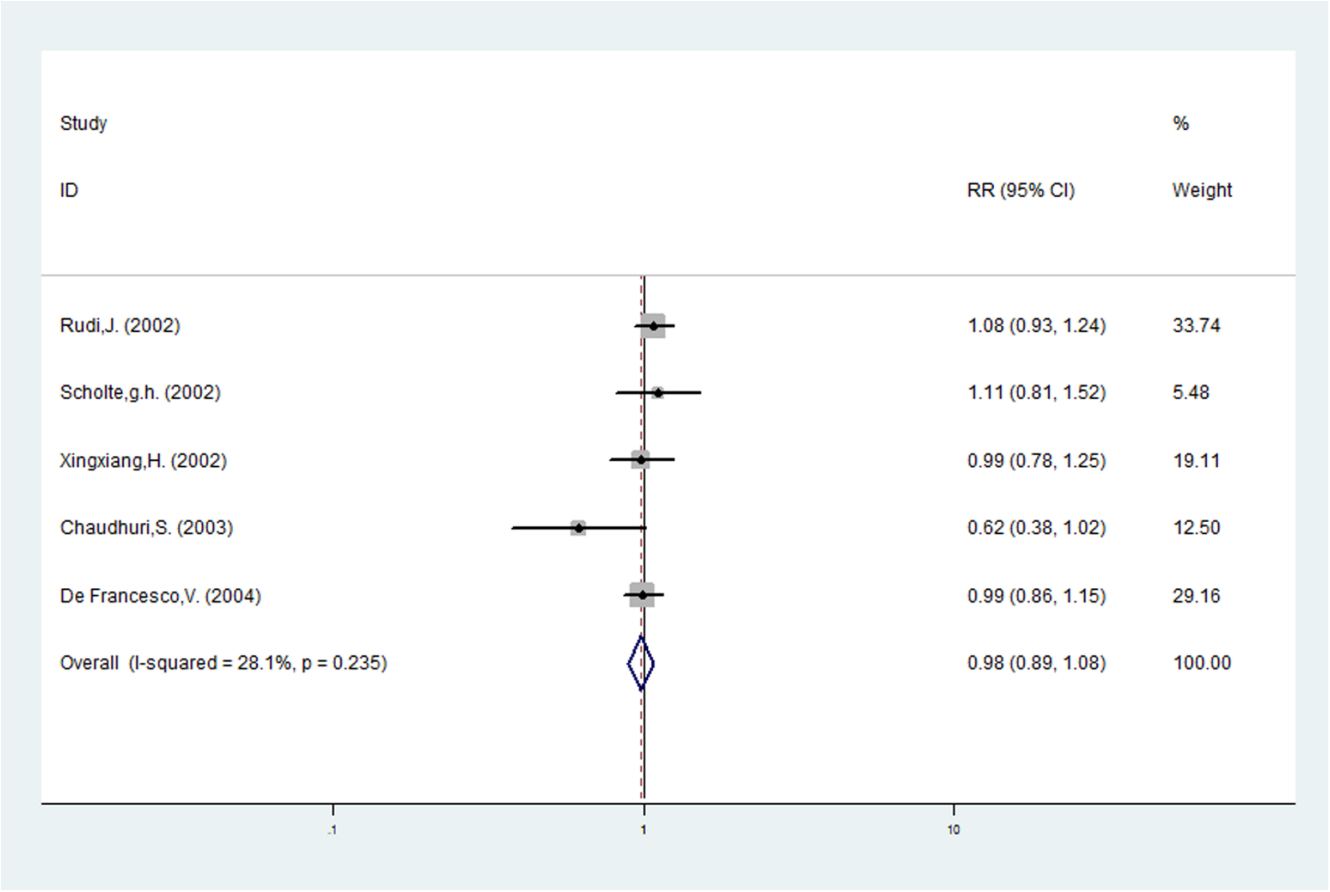
Forest plot of the association between *vacA* m1 and *vacA* m2 and eradication of *H*. *pylori*.

Risk ratios regarding the effects of *vacA* m1 and *vacA* m2 genotypes on *H*. *pylori* eradication rates were available for all five trials, which included data from 391 patients (135 patients in the *vacA* m1 group and 256 patients in *vacA* m2 group). A fixed-effects model was used because significant heterogeneity was not present (I^2^ = 28.1%, *P* = 0.235). The pooled *H*. *pylori* eradication rate was 84% (95%CI: 71–97%) for *vacA* m1 and 84% (95%CI: 80–89%) for *vacA* m2 (Figs 5 and 6). The pooled RR was 0.981 (95%CI: 0.891–1.080, *P* = 0.690; Table 2 and Fig 7). Based on these results, we determined that there was no statistically significant difference in *H*. *pylori* eradication rates between *vacA* m1 and *vacA* m2 genotypes based on therapy. Similarly, subgroup analysis based on region, in European or Asian, indicated that there was no statistically significant difference in *H*. *pylori* eradication rates between *vacA* m1 and *vacA* m2 strains (RR: 1.045, 95%CI: 0.949–1.151, *P* = 0.372; RR: 0.819, 95%CI: 0.508–1.322, *P* = 0.414; Table 2).

### Association between *cagA* status and eradication of *H*. *pylori*

**Fig 8:**
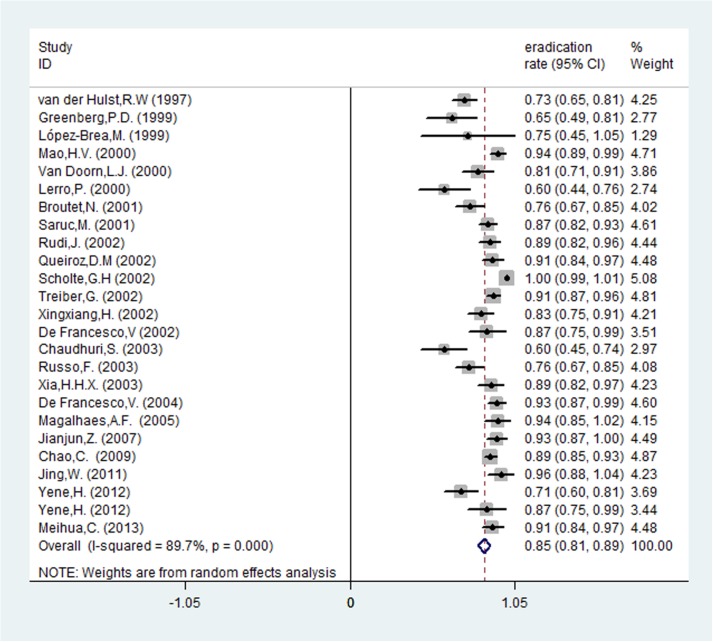
The pooled eradication rate of *H*. *pylori* with *cagA*-positive.

**Fig 9:**
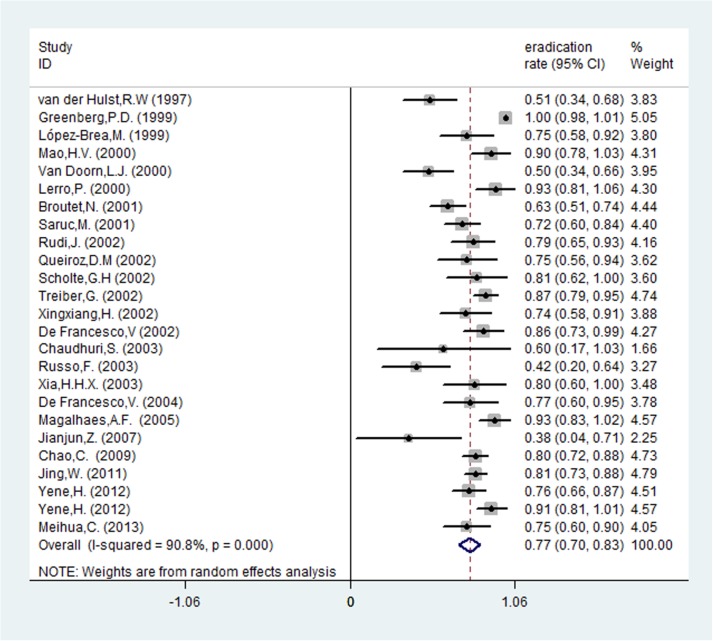
The pooled eradication rate of *H*. *pylori* with *cagA*-negative.

**Fig 10:**
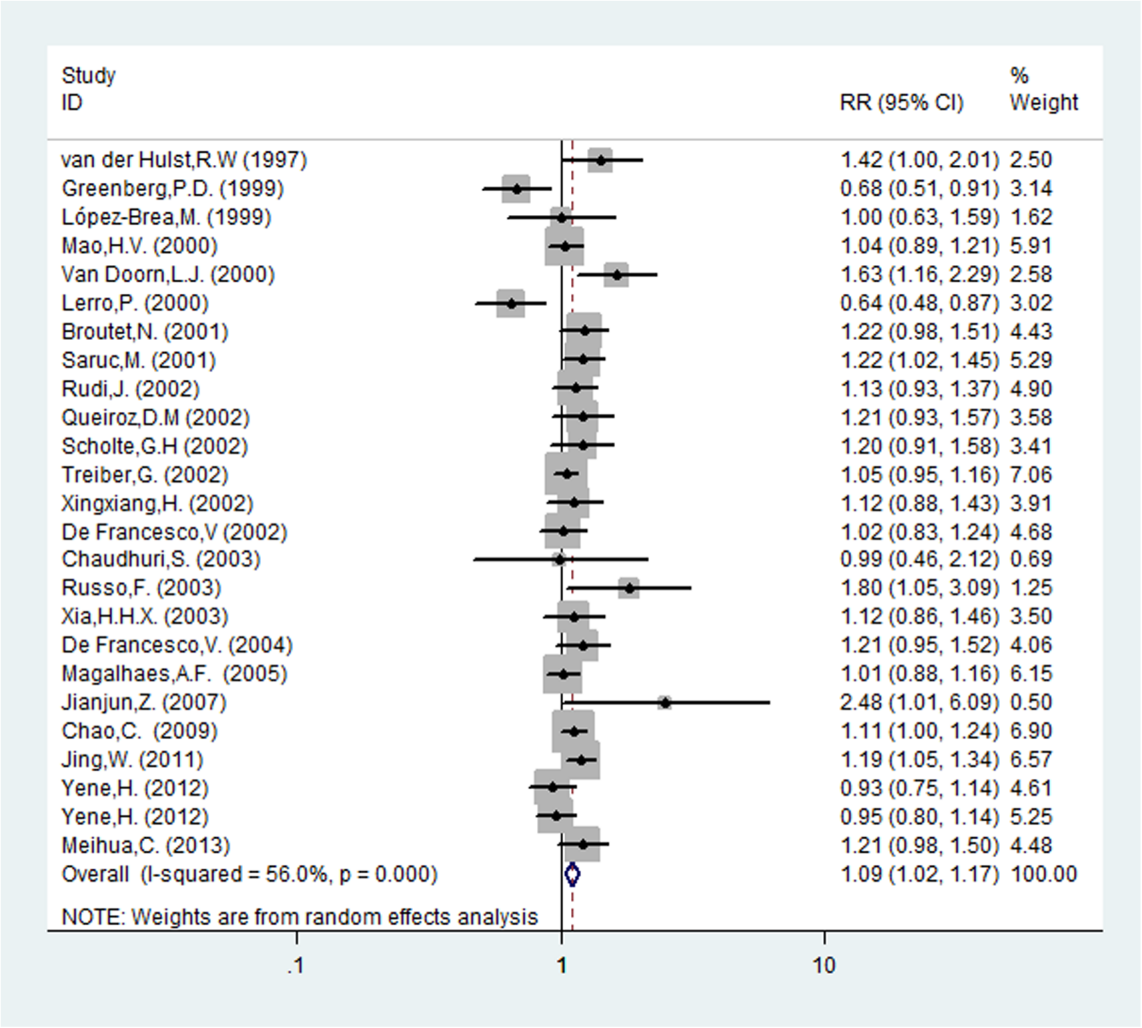
Forest plot of the association between *cagA*-positive and *cagA*-negative and eradication of *H*. *pylori*.

Risk ratios regarding the effects of *cagA*-positive and *cagA*-negative status on *H*. *pylori* eradication rates were available for all 25 trials, which included data from 2693 patients (1793 patients in the *cagA*-positive group and 900 patients in *cagA*-negative group). The pooled *H*. *pylori* eradication rate was 85% (95%CI: 81–89%) for *cagA*-positive and 77% (95%CI: 70–83%) for *cagA*-negative patients (Figs 8 and 9). We found that eradication rates were higher by approximately 8% in the *cagA*-positive compared with the *cagA*-negative group and that the pooled RR was 1.094 (95%CI: 1.025–1.168, *P* = 0.007; Table 2 and Fig 10). There was significant heterogeneity that existed among studies (I^2^ = 56.0%, *P*<0.001). To further investigate the sources of heterogeneity, we conducted a sensitivity analysis. After removing the most obvious outlying study by Van Doorn et al. (RR: 1.63) [8], heterogeneity remained (I^2^ = 51.7%, *P* = 0.002). In the remaining studies, using a random-effects model, we still concluded that *cagA*-positive strains had higher *H*. *pylori* therapy eradication rates compared with *cagA*-negative ones (RR: 1.083, 95%CI: 1.017–1.153, *P* = 0.013).

Next, we conducted subgroup analyses based on region (Europe, Asia or South America), disease ((PUD) or NPUD), detection method of eradication (polymerase chain reaction (PCR), protein chip, enzyme-linked immunosorbent assay (ELSIA) or western blot (WB)) and therapeutic regimen (dual-, triple-, quadruple- or sequential therapy). Regional subgroup analysis showed that for Europe and Asia, pooled RRs were 1.138 (95%CI: 1.000–1.295, *P* = 0.049) and 1.118 (95%CI: 1.051–1.190, *P*<0.001), respectively, regarding the effect of *cagA*-positive compared with *cagA*-negative genotype on eradication. However, in South America, *cagA*-positive strains had similar *H*. *pylori* therapeutic rates compared with *cagA*-negative strains (RR: 1.104, 95%CI: 0.953–1.279, *P* = 0.186; Table 2). Disease subgroup analysis showed that PUD and NPUD subgroups did not improve eradication rates (RR: 1.274, 95%CI: 0.664–2.445, *P* = 0.467; RR: 0.988, 95%CI: 0.861–1.134; *P* = 0.865; Table 2). Subgroup analysis based on the detection method of eradication showed that the *cagA*-positive genotype was associated with higher eradication rates in the PCR and protein chip subgroups (RR: 1.232, 95%CI: 1.142–1.329, *P*<0.001; RR: 1.200, 95%CI: 1.060–1.359, *P* = 0.004; Table 2) but not in the ELSIA or WB subgroups (RR: 1.048, 95%CI: 0.972–1.130, *P* = 0.223; RR: 0.801, 95%CI: 0.534–1.203, *P* = 0.285; Table 2). Therapeutic regimen subgroup analysis showed that the *cagA*-positive genotype was associated with higher eradication rates in the triple therapy (RR: 1.090, 95%CI: 1.006–1.181, *P* = 0.034) but not in dual- (RR: 0.978, 95%CI: 0.414–2.307, *P* = 0.959), quadruple- (RR: 1.134, 95%CI: 0.946–1.360, *P* = 0.173) or sequential therapy subgroups (RR: 1.114, 95%CI: 0.997–1.244, *P* = 0.057; Table 2).

### Publication bias

We performed the Begg’s and Egger’s tests to quantitatively evaluate the publication bias of the association between *vacA* and *cagA* for the successful eradication of *H*. *pylori* infection. Publication bias observed in this meta-analysis was not significant. Detailed information for the publication bias test is summarized in Table 3.

**Table 3:** Publication bias.

Genotype	Begg's test		Egger's	
	z-value	p value	t-value	p value
***vacA*s1/s2**	1.36	0.175	1.81	0.113
***vacA*m1/m2**	0.24	0.806	-1.16	0.33
***cagA*+/-**	1.05	0.293	1.45	0.16

## Discussion

In this meta-analysis study, the effect of virulence factors *vacA* and *cagA* on eradication treatment was analyzed systematically and the cumulative eradication rates were calculated. Our results showed that the eradication rates in patients infected with *vacA* s1 and s2 strains were 83% and 73%, respectively, for *cagA*-positive, and 85% and 77%, respectively, for *cagA*-negative. Patients with *vacA* s1 and *cagA*-positive strains were more likely to be eradicated, irrespective of *vacA* m subtype. In addition, the correlation of virulence factors with *H*. *pylori* eradication was also affected by factors such as region, detection method of eradication and therapeutic regimen. Our study provides useful information regarding the prediction of eradication outcome and for exploring molecular mechanisms of bacterial resistance.

VacA and CagA are the most intensively studied pathogenic factors of *H*. *pylori*. It is generally accepted that the *vacA* s1 and *cagA*-positive strains are likely more virulent and more closely related with gastric diseases. Our meta-analysis showed that strains carrying more virulence factors were more likely to be eradicated than other strains. We speculated the following reasons for this phenomenon: 1. In patients with a *vacA* s1, *cagA*-positive infection the inflammatory cell infiltration was significantly higher than in those with *vacA* s2, *cagA*-negative [21]. On the one hand, inflammatory factors can regulate gastric acid secretion; on the other hand, inflammation-related cytokines can increase local blood flow, which is conducive to antibiotic spread. Changes in gastric acid secretion and improvement in local blood flow can affect the delivery of antibiotics. 2. In patients with a *vacA* s1, *cagA*-positive infection, the permeability of drugs in the gastric mucosa may be significantly higher than in those who were *vacA* s2, *cagA*-negative. *VacA s1*, *cagA*-positive strains can cause more severe mucosal damage, which may allow better penetration of antibiotics from the gastric lumen [22] and allow better systemic delivery of drugs [23]. 3. One study reported that *H*. *pylori* density is higher and growth is faster in patients with a *vacA* s1, *cagA*-positive infection relative to *vacA* s2, *cagA*-negative patients. In addition, antibiotics have a stronger bactericidal effect during proliferation. 4. *VacA* s1, *cagA*-positive strains promoted synergistic increases in *H*. *pylori* eradication. *VacA* s1 strains have a significant correlation with the presence of *cagA* [7]. Most *cagA*-positive strains are *vacA* s1, and *cagA*-negative strains are *vacA* s2. Thus, it is possible that these two factors together improve the eradication effect. 5. Infection with high virulence *H*. *pylori* is more susceptible to bacterial resistance-related gene mutations. For example, some researchers have found that *vacA* s1- and *cagA*-positive strains often contained the A2143G mutation. Such mutations are associated with bacterial clarithromycin resistance [24]. In 2006, Suzuki et al.[25] performed a meta-analysis to compare the eradication efficacy of CagA-positive and CagA-negative strains in 14 articles. In the present study, we conducted an updated, detailed meta-analysis of 25 published papers to further confirm their study. We also calculated the pooled eradication rate of *H*. *pylori* and evaluated the cumulative RR. Our results indicate that eradication rates were greater by approximately 8% in the *cagA*-positive group compared with the *cagA*-negative group and that the pooled RR was 1.094.

In a further subgroup analysis, the relationship between virulence factor status and eradication efficacy was stratified based on region, disease, detection method of eradication and therapeutic regimen. The results showed that in Europe and Asia, the eradication efficacy was better for *vacA* s1, *cagA*-positive compared with *vacA* s2 *cagA*-negative strains. However, in South America there was no significant difference in the eradication outcome of patients infected with *cagA*-positive or *cagA*-negative strains. These results suggest that in Europe and Asia, patients infected with the *vacA* s1 and *cagA*-positive strains, despite an increased risk of stomach disease, can achieve better eradication rates. In South America, owing to the relatively small number of included studies (n = 1), it cannot be concluded whether *vacA* s1/s2 subtypes were associated with eradication. In the stratified analysis of therapy regimens, *vacA* s1 status had higher eradication rates in the triple-therapy patients. Because of the small number of included studies, we cannot make a comparison between sequential- and quadruple therapy. *cagA*-positive had higher eradication rates in patients who received the triple therapy but not in those who received dual-, quadruple- or sequential therapies. This shows that the efficacy of quadruple or sequential therapy is not affected by *cagA* virulence factors. Perhaps this is one reason why quadruple- or sequential therapy was used more gradually as the first-line treatment than triple therapy. The source of disease analyses showed that neither in the PUD nor in the NPUD subgroups were eradication rates improved regarding *cagA* status, indicating that the relationship between *cagA* and eradication efficacy is not affected by disease status. In addition, when PCR and protein chip methods were used to determine eradication rates, the *cagA*-positive eradication rate was higher than the *cagA*-negative one. However, the same conclusion cannot be reached when the ELISA or WB methods were used. It is possible that compared with the ELISA and WB methods, PCR and protein chip may be more sensitive, reliable and accurate. The traditional ELSIA and WB methods are quantitative assays for detecting serum cagA antibody levels and the results may be affected by various factors. Although the protein chip is also based on the detection of protein, only a small amount of protein sample is needed, because sensitivity is 100 times greater than that of the WB and ELISA, so the results are more reliable and accurate. PCR is a qualitative analysis of the virulence factor at the gene level in gastric mucosal tissues and formalin-embedded specimens. Even if little DNA is contained in these specimens, virulence factors can also be detected using PCR amplification, which can reflect the real situation of gastric infection. In addition, PCR detection also avoids the time-consuming and harsh conditions of *H*. *pylori* culture, so the PCR method will have a broader application in the future.

This meta-analysis had some limitations. First, we only included studies written in English or Chinese. Thus, selection bias might exist. Second, some of individual studies may not result in a tangible conclusion due to PPI brand, type of antibiotic, the small sample size, regional differences, treatment regimen differences, disease background, or differences in detection methods. Therefore, the results from this meta-analysis should be verifiable by conducting a larger and thorough study. Third, the articles on *cagA* and eradication efficacy included two with children as subjects and the remainder with adults. Differences between children and adults may have a potential impact on the eradication efficacy. Fourth, we only analyzed the relationship between *cagA*-positive or negative and eradication efficacy, and did not explore completeness of cag PAI and eradication efficacy. cag PAI is a 40-kb DNA fragment found in the *cagA*-positive strain. The integrity of cag PAI is different in different *H*.*pylori* strains and can be divided into three types: intact-PAI, partially deleted-PAI, and totally deleted-PAI genes. According to the structural differences of cag PAI, *H*. *pylori* can be divided into different virulent groups and cause different clinical outcomes after infection. Because there were few reports regarding differences in integrity of cag PAI and drug resistance, we were unable to perform a meta-analysis on this. In addition, polymorphisms in the EPIYA sequence determine differences in cagA protein function. Based on the EPIYA motifs, *H*. *pylori* was subcategorized as Western or East Asian strains. However, until now there has been no study that has investigated its relationship with eradication efficacy. These unresolved problems are critical in exploring the relationship between virulence factors and eradication efficacy and will be the focus of future research. Fifth, the study of *vacA* and *H*. *pylori* eradication is mainly focused on s and m regions; therefore, a meta-analysis of only these regions was carried out in our study. In fact, polymorphisms of *vacA* mainly include three areas: s, i, and m. However, the relationship between the i region and *H*. *pylori* eradication has been reported to a lesser extent. Only one study has shown that low virulence *vacA* i2 is related to A2143G mutations, and high virulence *vacA* i1 is related to A2142G mutations [26], suggesting that there may be some relationship between *vacA* i genotypes and antibiotic resistance. More research is still needed to further validate this correlation. However, the number of such studies is too small to be sufficient for meta-analysis. Sixth, this meta-analysis only summarizes the relationship between virulence factors and drug resistance in patients infected with a single strain. In the included studies used in this meta-analysis, only the one by Russo mentioned two cases of mixed infection of *vacA* s1 and s2, one of which was successfully treated while the other was not. In the rest of the included studies, authors had detected the presence of mixed infections, but they did not analyze the relationship between mixed infection and *H*. *pylori* eradication. Therefore, we cannot further analyze the relationship between mixed infection and eradication efficacy. However, the actual relationship between mixed infection and *H*. *pylori* eradication, and whether it is easier or more difficult to eradicate than a single infection, still requires further research in this area, especially in regions with a high proportion of mixed infections. Seventh, in our included data, the eradication rate was calculated using PP analysis. This method, removing the failed to complete test subjects, will inevitably result in an overestimation of the eradication rates. Because of the small number of studies using intent-to-treat (ITT) analysis, it is not possible to compare the effects of these two analysis programs on eradication rates. Eighth, other virulence factors of *H*. *pylori*, such as *dupA*, *oipA* and *iceA*[27–30]may also affect eradication outcomes. However, relatively few of these factors have been reported in the literature, which did not allow us to conduct a systematic meta-analysis of them.

## Conclusion

In summary, this meta analysis demostrateded virulence factors *vacA* s1 and *cagA* indeed affect the eradication efficacy of *H*. *pylori*. *vacA* s1, *cagA*-positive strains are easier to eradicate in infected patients, but this has nothing to do with the *vacA* m subtype. In addition, the correlation between *vacA* s1, *cagA*-positive and eradication efficacy was also affected by region, detection method of eradication and therapeutic regimen. Our results suggest that although *vacA* s1 and *cagA*-positive strains are high-risk factors for the development of gastric diseases, the eradication efficacy is better than the other *H*. *pylori* strains. In patients infected with *vacA* s2 and *cagA*-negative strains, though less pathogenic, *H*. *pylori* is more difficult to eradicate. This may be one of the causes of *H*. *pylori* antibiotic resistance. Our study may complement investigations of resistance-related bacterial factors, providing possible clues to further explore *H*. *pylori* antibiotic resistance, which may help in finding a new therapeutic target to eradicate *H*. *pylori*. For all that, the results from our meta-analysis should be verifiable by conducting a larger and thorough study in the further.

## Supporting information

S1 Table PRISMA checklist. (DOC)

S2 Table Results of Newcastle–Ottawa scale (NOS) assessment for the included studies. (DOCX)
