# Supplementary material for: The association between vacA or cagA status and eradication outcome of Helicobacter pylori infection: A meta-analysis
Source: PLoS One. 2017 May 11;12(5):e0177455. doi: 10.1371/journal.pone.0177455 (PMC5426689; doi:10.1371/journal.pone.0177455)
Supplement: S2 Table — (DOCX) [file pone.0177455.s002.docx]

S2 Table. Results of Newcastle – Ottawa scale (NOS) assessment for the included studies

| Author | Year | Selection | Comparability | outcome | Total NOS star rating |  |  |
| --- | --- | --- | --- | --- | --- | --- | --- |
| **For *vacA* s1/s2** | |  |  |  |  |  |  |
| López-Brea,M. | 1999 | 4 | 0 | 3 | 7 |  |  |
| Van Doorn,L.J. | 2000 | 4 | 0 | 3 | 7 |  |  |
| Rudi,J. | 2002 | 4 | 0 | 3 | 7 |  |  |
| Scholte,G.H. | 2002 | 3 | 1 | 3 | 7 |  |  |
| Xingxiang,H. | 2002 | 4 | 1 | 3 | 8 |  |  |
| Chaudhuri,S. | 2003 | 3 | 1 | 3 | 7 |  |  |
| Russo,F. | 2003 | 4 | 1 | 3 | 8 |  |  |
| De Francesco,V. | 2004 | 4 | 1 | 3 | 7 |  |  |
| Shengli,N. | 2014 | 3 | 1 | 3 | 7 |  |  |
| **For *vacA* m1/m2** | |  |  |  |  |  |  |
| Rudi,J. | 2002 | 4 | 0 | 3 | 7 |  |  |
| Scholte,G.H. | 2002 | 3 | 1 | 3 | 7 |  |  |
| Xingxiang,H. | 2002 | 4 | 1 | 3 | 8 |  |  |
| Chaudhuri,S. | 2003 | 3 | 1 | 3 | 7 |  |  |
| De Francesco,V. | 2004 | 3 | 1 | 3 | 7 |  |  |
| **For *cagA*+/-** | |  |  |  |  |  |  |
| van der Hulst,R.W | 1997 | 4 | 1 | 3 | 8 |  |  |
| Greenberg,P.D. | 1999 | 3 | 1 | 3 | 7 |  |  |
| López-Brea,M. | 1999 | 4 | 0 | 3 | 7 |  |  |
| Mao,H.V. | 2000 | 3 | 1 | 3 | 7 |  |  |
| Van Doorn,L.J. | 2000 | 4 | 0 | 3 | 7 |  |  |
| Lerro,P. | 2000 | 3 | 1 | 3 | 7 |  |  |
| Broutet,N. | 2001 | 3 | 1 | 3 | 7 |  |  |
| Saruc,M. | 2001 | 3 | 1 | 3 | 7 |  |  |
| Rudi,J. | 2002 | 4 | 0 | 3 | 7 |  |  |
| Queiroz,D.M | 2002 | 4 | 1 | 3 | 8 |  |  |
| Scholte,G.H | 2002 | 3 | 1 | 3 | 7 |  |  |
| Treiber,G. | 2002 | 4 | 1 | 3 | 8 |  |  |
| Xingxiang,H. | 2002 | 4 | 1 | 3 | 8 |  |  |
| De Francesco,V | 2002 | 3 | 1 | 3 | 7 |  |  |
| Chaudhuri,S. | 2003 | 3 | 1 | 3 | 7 |  |  |
| Russo,F. | 2003 | 4 | 1 | 3 | 8 |  |  |
| Xia,H.H.X. | 2003 | 3 | 1 | 3 | 7 |  |  |
| De Francesco,V. | 2004 | 3 | 1 | 3 | 7 |  |  |
| Magalhaes,A.F. | 2005 | 4 | 0 | 3 | 8 |  |  |
| Jianjun,Z. | 2007 | 3 | 1 | 3 | 7 |  |  |
| Chao,C. | 2009 | 4 | 0 | 3 | 7 |  |  |
| Jing,W. | 2011 | 4 | 0 | 3 | 7 |  |  |
| Yene,H. | 2012 | 3 | 1 | 3 | 7 |  |  |
| Yene,H. | 2012 | 3 | 1 | 3 | 7 |  |  |
| Meihua,C. | 2013 | 4 | 1 | 3 | 8 |  |  |
